# 

**Published:** 1888-02

**Authors:** 


CONTENTS

VOL. 35 NO. 2.

| | PAGE. |
|---|---|
| Special | 25 |
| William Eglinton | 25 |
| A Buddhist Missionary | 30 |
| The Castor Oil Plant | 33 |
| The Tobacco Habit | 34 |
| Psychography | 35 |
| Psychometry as a Means of Diagnosis | 37 |
| The Habit of Kissing | 38 |
| Little Things that Kill | 39 |
| A Mathematical Prodigy | 40 |
| Is Romanism Overwhelming Us | 40 |
| The Largest Statue in the World | 41 |
| French Toilet Preparations | 42 |
| Good Rules for Winter | 42 |
| Mind Cure Convention | 43 |
| Book Notices | 44 |
| Household | 45 |
| Miscellaneous | 47 |

INDEX TO ADVERTISEMENTS OF HALF A PAGE AND OVER.

| | PAGE. |
|---|---|
| Crystal Pepsin Tablets | I |
| Zylonite | II |
| Stevensville Mills | II |
| Hollow Suppositories | III |
| Scientific Truth | IV |
| George Stinson & Co | VI |
| Lactopeptine | VII |
| Deadly Drinking Water | VIII |
| Telegraph Medicine Co | X |
| Dr. Molesworth | X |
| J. B. Watkins' Land Mortgage Co | X |
| Bovinine | XI |
| Celerina | XIII |
| Premiums of Books | XIV |
| Premiums of Genuine Silver Plate | XV |

